# Potential Role of MicroRNA-210 as Biomarker in Human Cancers Detection: A Meta-Analysis

**DOI:** 10.1155/2015/303987

**Published:** 2015-09-13

**Authors:** Jiongjiong Lu, Feng Xie, Li Geng, Weifeng Shen, Chengjun Sui, Jiamei Yang

**Affiliations:** Department of Special Treatment, Eastern Hepatobiliary Surgery Hospital, Second Military Medical University, No. 225, Changhai Road, Shanghai 200438, China

## Abstract

We conducted this meta-analysis aimed to evaluate diagnostic accuracy of miR-210 in human cancers. A total of 673 cancer patients and 606 cancer-free individuals from 13 studies were contained in this meta-analysis. The overall diagnostic results in our study showed that the pooled sensitivity was 0.70, specificity was 0.76, and the AUC was 0.80. In addition, the PLR and NLR were 2.9 and 0.39, with DOR of 8. After the outliner exclusion detected by sensitivity analysis, these parameters had minimal change, which confirmed the stability of our work. The results in our studies showed that the miR-210 assay yielded relatively moderate accuracy in cancer patients and cancer-free individual differentiation. More basic researches are needed to highlight its role as supplement in clinical treatment.

## 1. Introduction

Cancer, with an estimate of millions of deaths each year, is considered as one of the highest mortalities worldwide [1–3]. The complex and progressive molecular progress involved in cancer development made it a challenge in clinic, bringing the early stage treatment to the front as it seems easier to control the disease. For example, 5-year survival rate is approximately 98% for renal cancer stage I patients, while survival drops to 50% for patients in stage III [4, 5]. For instance, 5-year survival rate of 80% for stage I but only 10% for stage IV patients with lung cancer also accounts for the importance of early detection [6, 7]. Thus, the most effective way to improve the disease outcomes and therefore reduce the worldwide health burden is the development of diagnostic tool for early detection.

Nowadays, the gold standard for cancer detection is the histological evaluation of biopsy. Though it is the most reliable way in cancer prediction with relatively high sensitivity and specificity, its usage is still restricted in clinic for the suffering of patients resulting from the invasive nature [8]. Several currently blood-based biomarkers may enhance the early cancer detection without the unpleasant procedure, including carcinoembryonic antigen (CEA), carbohydrate antigen 19-9, alphafetoprotein (AFP), and prostate specific antigen (PSA), but the low accuracy makes them minimally useful for the supplement of existing screening methods [9–12]. Therefore, although the diagnostic tool for early cancer detection could reduce the mortality, the effective biomarkers are still absent.

The discovery of microRNAs (miRNAs), a group of regulatory RNAs with 22 nucleotides in length, has opened up a new field in molecular diagnosis of cancer at early stage [13]. miRNAs have proven to be involved in the initiation and progression of human malignancy by influencing the degradation or translation of hundreds of mRNAs [13–15]. Further, their abnormally expression levels are found to be associated with a variety of diseases, including pancreatic cancer, lung cancer, and breast cancer [16–18]. What is more, miRNAs, present in human body matrix like plasma, sputum, feces, and serum, are resistant to RNase activity and keep stable even in extreme environment, which is the evidence of its stability [19, 20]. For instance, reproducibility is another advantage of miRNAs as they are stable and easy to be accessed by quantitative reverse transcription polymerase chain reaction (qTR-PCR) methods [21, 22]. Therefore, miRNAs may be the promising candidate as invasive biomarkers for early cancer detection.

MicroRNA-210 (miRNA-210, miR-210), a member of miRNAs, has been largely studied in the past several years and has been identified as a major induced miRNA under hypoxia [23, 24]. Thus, unusual expression of hypoxia-inducible miR-210 may link to cancer, as hypoxia is a common feature of the neoplastic microenvironment [25]. Since Wang et al. firstly demonstrated the miR-210 might have a prediction value for pancreatic cancer with sensitivity 0.42 and specificity 0.73, more researches have been done to explore the possible clinical usage of miR-210 [16, 26–28]. For example, Anjuman et al. found that miR-210 were present in considerably higher levels in sputum of lung cancer patients than cancer-free individuals and yielded diagnostic accuracy of 0.77 in lung cancer detection [28]. For instance, the improvement in diagnostic performance of miR-210 with sensitivity 0.84 and specificity 0.82 in the diagnosis of breast cancer was pronounced by Madhavan et al., which lightens the potential value of miR-210 with relatively better accuracy in supplement of the current screening tools [29]. Though other single studies as well investigated the important diagnostic role of miR-210 in various cancers, the limited sample size, different study design, and lack of unified standard resulted in conflicting results. And notably two meta-analyses have already been conducted to evaluate the performance of miR-210 as a prognostic factor in breast cancer, but the there is no meta-analysis focusing on the diagnostic value of miR-210 and systematically pooling all the relative published studies of miR-210 in a series of cancers [30, 31]. Thus, we performed the present meta-analysis to summarize the overall accuracy of miR-210 in cancer detection and further identify its value in clinical use.

## 2. Methods

### 2.1. Search Strategy

We conducted a literature research in database including PubMed, EMBASE, CNKI, Wan Fang library before August 6, 2014, in order to identify the relevant records about miR-210 in cancer. The key words we used in the research were “cancer” or “tumor” or “neoplasm” or “malignancy” or “neoplasia” and “microRNA-210” or “miR-210” or “has-miR-210” and “sensitivity” or “specificity” or “ROC curve” or “accuracy.”

Two reviewers checked the abstract of the studies and read the full-text if necessary to identify the final included studies based on the following included criteria: (1) studies which evaluated the diagnostic value of miR-210 for detecting cancer, (2) case-control design with control group of benign disease or healthy people, and (3) studies providing sufficient data to calculate diagnostic parameters.

### 2.2. Data Extraction and Quality Assessment

The necessary information of the included studies was extracted by two reviewers and filled onto standardized data forms. The data extracted were (1) first author, (2) year of publication, (3) country, (4) ethnicity, (5) number, age, and male ratio of the case and control groups, (6) cancer type, (7) specimen, and (8) the diagnostic parameters including sensitivity and specificity. We also scored each of the included studies according to the QUADAS-2 (quality assessment of diagnostic accuracy studies-2) tool. With the max score of 7, the quality of the included studies can be judged by the results.

### 2.3. Statistical Analysis

The random-effects model was used in our analysis to summarize the sensitivity, specificity, and other parameters [37]. The SROC curve (summary receiver operating characteristic) and its under area AUC were also gathered to evaluate the accuracy of miR-210 in cancer [38]. In addition, we performed metaregression to investigate the heterogeneity between the included studies with *P* < 0.05 considered statistically significant [39]. Confirming the stability of our study, we also conducted the sensitivity analysis and further performed the outliner exclusion in our work. For instance, Deeks et al.'s funnel plot was employed to assess the potential publication bias [40]. All the statistical analyses were undertaken using Stata 12.0.

## 3. Results

### 3.1. Study Research

**Figure 1:**
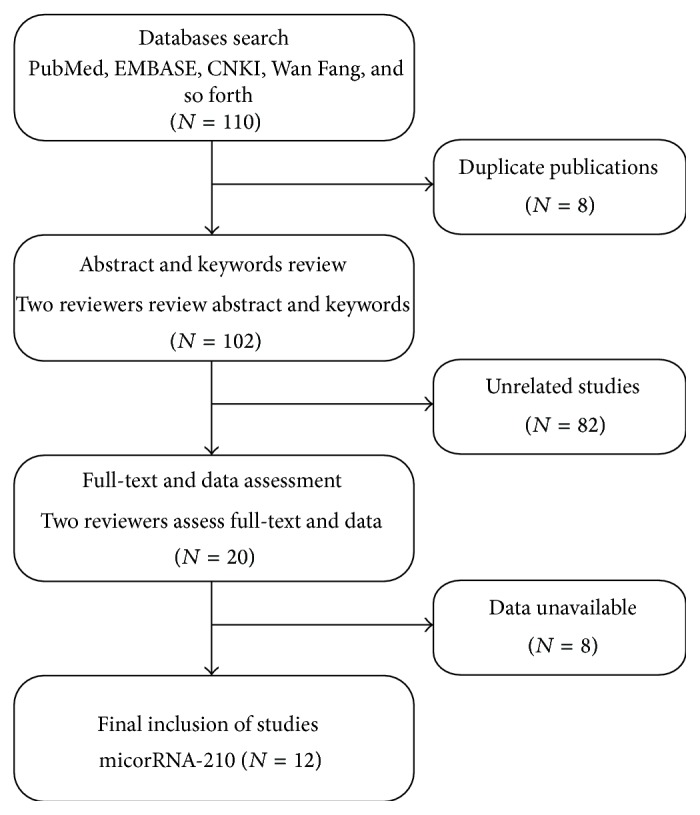
Flow diagram of publications research process.

110 manuscripts were identified from the initial search including PubMed, EMBASE, CNKI, and Wan Fang databases. After 8 records were excluded for duplications, totals of 102 records were left for the next step judgment. Then, 82 records were excluded as unrelated studies by reviewing the abstract and keywords. After full-text reading of the remaining 20 records, 8 of them were rejected due to the unavailable data. Thus, 12 records related to miR-210 in cancer detection were finally included in the meta-analysis [5, 16–18, 26–28, 32–36]. The flow diagram for literature research processes is shown in Figure 1.

The characteristics of studies included in this analysis are summarized in Table 1. 673 cancer patients and 606 cancer-free individuals from 13 studies published from 2009 to 2014 were contained in this meta-analysis. All the 13 studies tested miR-210s expression using qRT-PCR methods based on plasma (*n* = 5), sputum (*n* = 3), serum (*n* = 4), and fecal (*n* = 1). Six of the studies were conducted in Caucasian and African population, 4 of them conducted in Asian population, and 4 of them performed in Caucasian population. Among the 13 studies, 6 explore the association between miR-210 expression and lung cancer, 2 investigated that in breast cancer, and the other 5 focused on pancreatic cancer (*n* = 2), renal cancer (*n* = 2), and leukemia (*n* = 1). In addition, two reviews independently scored the included studies based on QUADAS-2 score system. All of them had relatively high quality with scores between 4 and 7 (Table 1), indicating the reliable foundation of our analysis.

### 3.2. Outcomes of miR-210 Assay

**Figure 2:**
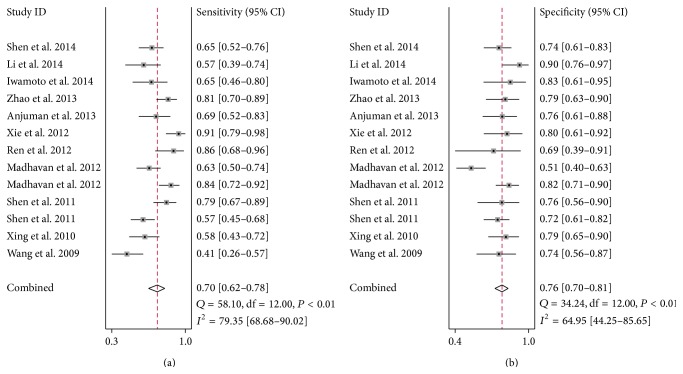
Forest plots of sensitivity (a) and specificity (b) of the overall 12 included publications.

**Figure 3:**
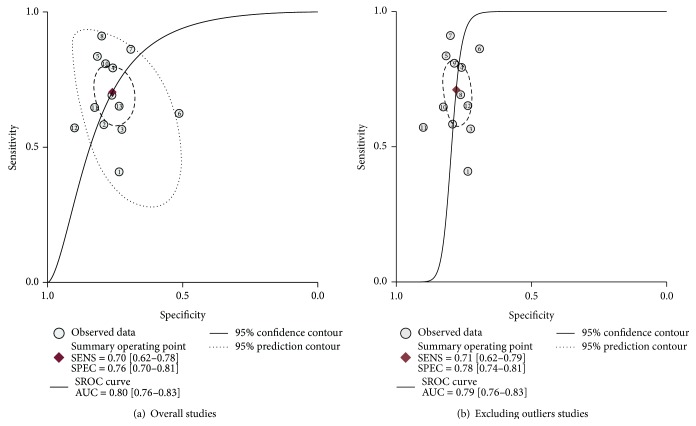
The SROC curves containing mean operating sensitivity and specificity point with AUC (a) overall and (b) after exclusion.

Considering the significant heterogeneity observed among the included studies (*I* ^2^ = 79.35% for sensitivity and *I* ^2^ = 64.95 for specificity, resp.) (Figure 2), the random-effect model was chosen in our analysis. As the SROC curve shown in Figure 3(a), the overall diagnostic results showed that the pooled sensitivity was 0.70 (95% CI: 0.62–0.78), specificity was 0.76 (95% CI: 0.70–0.81), and the AUC was 0.80 (95% CI: 0.70–0.83). The pooled positive likelihood ratios (PLR) and negative likelihood ratios (NLR) were also calculated by the bivariate meta-analysis with values of 2.9 (95% CI: 2.3–3.8) and 0.39 (95% CI: 0.29–0.52), respectively. The overall diagnostic odds ratio (DOR), ratio of PLR and NLR, was 8 (95% CI: 4–13). The results all together indicated a relatively moderate diagnostic accuracy of miR-210 in distinguishing cancer patients and cancer-free individuals.

### 3.3. Metaregression and Sensitivity Analyses

**Figure 4:**
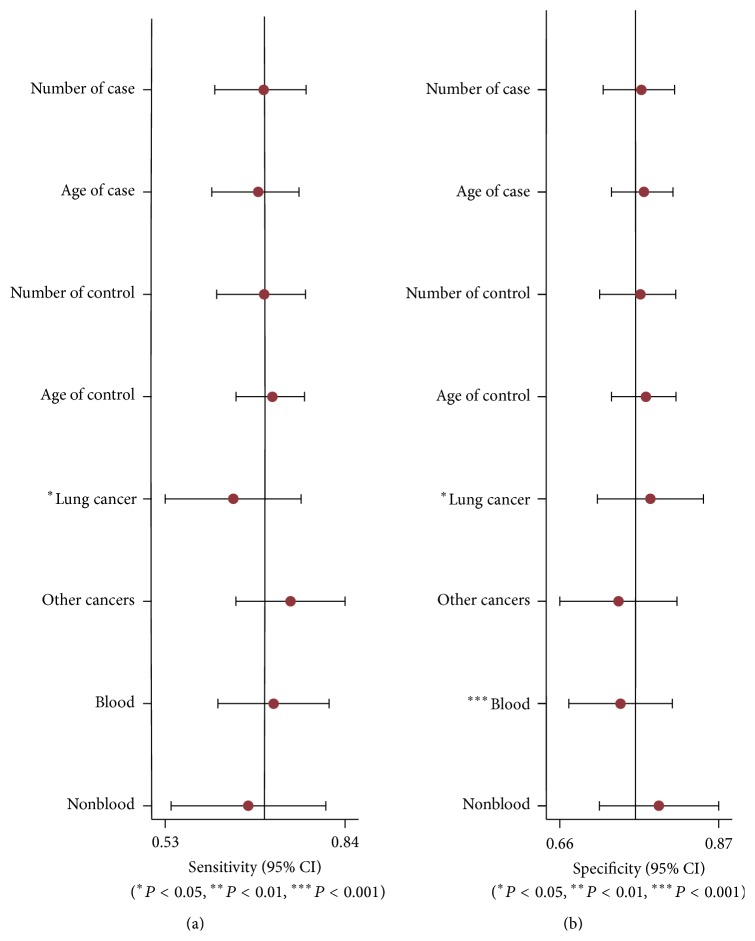
Multivariable metaregression (a) sensitivity and (b) specificity.

**Figure 5:**
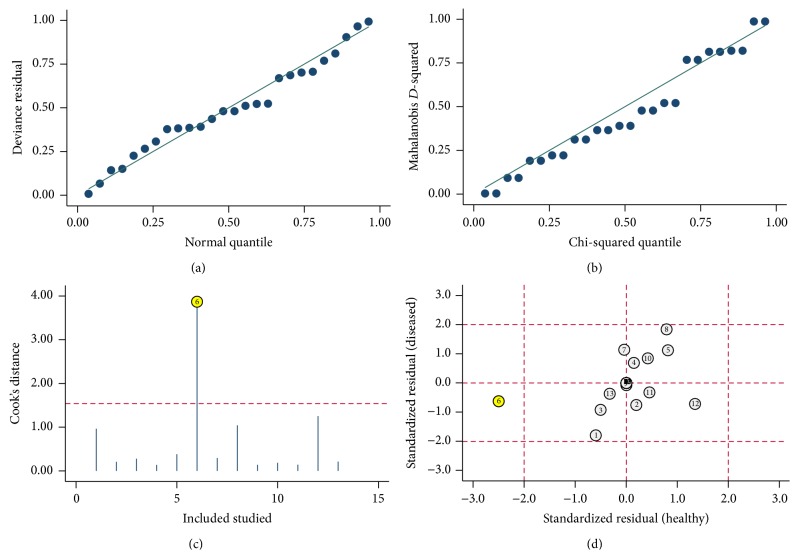
Influence analysis and outlier detection: (a) goodness of fit, (b) bivariate normality, (c) influence analysis, and (d) outlier detection.

In order to find potential sources of heterogeneity, we performed the metaregression based on the variables including number of case and control, age of case and control, cancer type, and specimen. The results in Figure 4 suggested that cancer type (*P* < 0.05) had an effect on sensitivity, while the cancer type (*P* < 0.05) and the specimen (*P* < 0.001) contributed to interstudy heterogeneity for specificity. We also conducted sensitivity analyses and further excluded 1 outliner found by influence analysis and outlier detection in Figure 5. After exclusion, the sensitivity increased from 0.70 to 0.71, specificity increased from 0.76 to 0.78, the PLR increased from 2.9 to 3.2, the NLR dropped from 0.39 to 0.37, DOR improved from 8 to 9, and AUC decreased from 0.80 to 0.79, showing minimal change with our overall analysis (Figure 3(b)). Combined with goodness of fit and bivariate normality analyses, we confirmed the robustness of our meta-analysis.

### 3.4. Publication Bias

**Figure 6:**
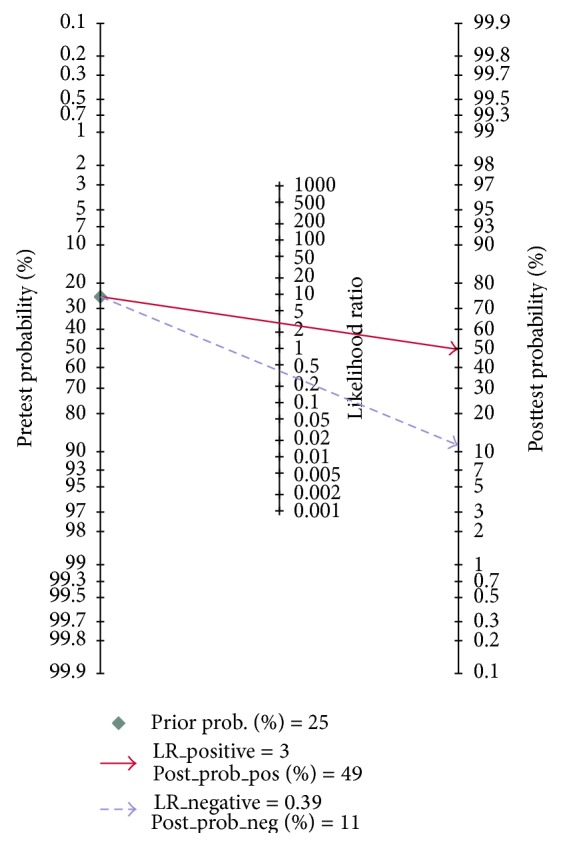
Fagan's nomogram in assessment of the test probabilities after miR-210 assay.

**Figure 7:**
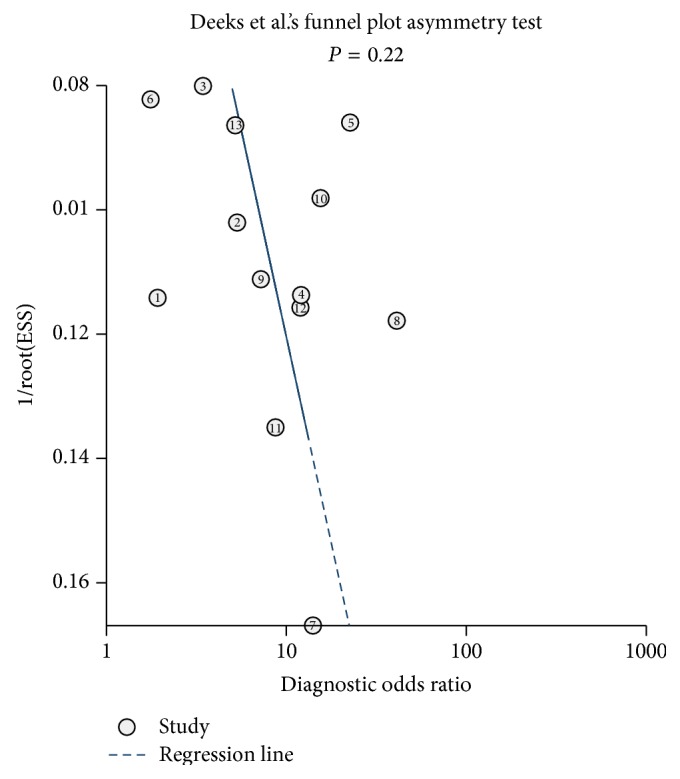
Deeks et al.'s funnel plots asymmetry test with regression line to explore publication bias.

Fagan's nomogram in Figure 6 describes the association between miR-210 assays results and the probability of cancer. For instance, when miR-210 assays were tested for any people with a pretest probability of 25% to have cancer, a positive result would improve posttest probability having cancer to 50%, while a negative result would drop the posttest probability to 12%. Thus, the miR-210 may serve as a noninvasive biomarker to supply the existing diagnostic methods. In addition, we conducted Deeks et al.'s funnel plot asymmetry test and found no significant publication bias in our study with *P* value of 0.22 (Figure 7).

## 4. Discussion

Cancer is a worldwide health problem due to the complex and progressive molecular procedure and the absence of effective diagnostic tool at cancer early stage [2]. Though the development of such invasive and effective biomarkers has been investigated for decades, little progress has been made until the discovery of miRNAs. miRNAs have been reported to associate with the development of tumor as a regulator in gene expression [13]. Large efforts have been made to investigate the link between abnormal miRNA expression and cancer, including the miR-210, the most consistently hypoxia-induced miRNA [41]. However, the diagnostic accuracy of miR-210 was inconsistent in literature due to the inescapable limitation of single study. Thus, we conducted the present meta-analysis to evaluate the diagnostic performance of miR-210 in cancer detection.

The pooled results in our study were sensitivity of 0.70 (95% CI: 0.62–0.78), specificity of 0.76 (95% CI: 0.70–0.81), and the AUC of 0.80 (95% CI: 0.70–0.83), indicating a moderate diagnostic efficiency of miR-210 in diagnosis of cancer. The pooled PLR and NLR were 2.9 (95% CI: 2.3–3.8) and 0.39 (95% CI: 0.29–0.52), respectively, with DOR of 8 (95% CI: 4–13), suggesting the relatively low level of miR-210 assay to identify or exclude cancer patients. Thus, due to the moderate accuracy, the application of miR-210 serving as a clinical biomarker still has a long way to go.

As the results in our analysis, a single miR-210 in cancer detection may lack sensitivity and specificity, but there are several areas we need to focus on in the future research in order to promote the usage of miR-210 in clinical treatment. Firstly, the mechanism of miR-210 abnormally expressed in cancer is not completely understood; more scientific and technological methods should be used in future basic research to provide better understanding of biological roles of miR-210 in cancer, hence lightening up the diagnostic value of miR-210. Recent studies suggested that hypoxic condition, which is a feature for solid tumor, may increase the level of miR-210 as miR-210 is related to the hypoxia-inducible factor- (HIF-) 1a and HIF-2a [41–43]. Although such connection of miR-210 and cancer highlights the function of miR-210 in cancer detection, the exact mechanism of it in tumor development needs further investigation.

Secondly, plenty of studies have demonstrated the advantages of multiple miRNAs combined assays, which may be the solution for the lack of accuracy of miR-210 in our analysis. For example, Shen et al. explored the prediction ability of miR-210 and miR-31 for lung cancer; combined use of the two miRNAs yielded 65.2% sensitivity and 89.7% specificity versus sensitivity of 67.2% and specificity of 31.5% of single miR-210 assay [36]. Not happening singly but in pairs, Anjuman et al. also found that single miR-210 test generated 0.77 accuracy in diagnosis of lung cancer, while the combined analysis of miR-210 and miR-31 had a better overall diagnostic performance with 0.83 [28]. For instance, we know that single miR-210 can cover a broad spectrum of cancers and the combination of miR-210 and other miRNAs may contribute to the accuracy improvement, but the combination way, as well as the unique group for specific cancer, needs to be further clarified.

Thirdly, although the ethnicity is not the source of heterogeneity according to the metaregression in the present analysis, cancer prediction based on population is still an important task in the future as different ethic patient may have specific characteristics of their tumors. What is more, the sample size was too small in our study with only 3 studies focused on the miR-210 expression in cancer in Asian population and no study explored the miR-210 function in only African populations, which resulted in unavoidable limitations. Actually, the included studies showed that the serum-based miR-210 assay in renal cancer yielded 81% sensitivity and 79.4% specificity in Caucasian populations but 65% sensitivity and 83% specificity in Asian populations [5, 34]. Thus, more fundamental research with long follow-up period should pay attention to the heterogeneity of miR-210 in cancer based on populations.

Fourthly, data normalization is currently a problem we need to deal with. For example, when we demonstrated that miR-210 was highly expressed in cancer, infeasible comparison can be done between studies as no reference substance can be found in the existing included studies, such as a miRNA sharing the same expression in cancer patients and cancer-free individuals. In addition, the cut-off values of miR-210 were varied in different studies and different cancers, which may result in the higher accuracy from lower cut-off value. Therefore, the standard should be set up in order to avoid the systemic differences.

## 5. Conclusion

In conclusion, the results in current meta-analysis showed that the application of miR-210 as the first-line screening tool in clinical treatment was immature due to lack of accuracy. However, the miR-210 assay showed potential used as a supplement for the existing diagnostic methods to improve accuracy. What is more, future research should focus on the combined usage of miR-210 with other miRNAs and make improvement in technic consensus such as data normalization.

## Figures and Tables

**Table 1:** Characteristics of the included studies.

First author	Year	Country	Ethnicity	Sample size		Male		Cancer	Specimen	Diagnostic power				QUADAS
				Control	Case	Control	Case			TP	FP	FN	TN	
Wang [16]	2009	USA	Caucasian	44	34	0.51	n.a.	Pancreatic cancer	Plasma	18	9	26	25	4
Xing [17]	2010	USA	Caucasian/African	48	48	0.68	0.54	Lung cancer	Sputum	28	10	20	38	5
Shen [18]	2011	USA	Caucasian/African	76	80	0.55	0.63	Lung cancer	Plasma	43	22	33	58	5
Shen [26]	2011	USA	Caucasian/African	58	29	0.68	0.66	Lung cancer	Plasma	46	7	12	22	6
Madhavan [32]	2012	Germany	Caucasian	61	76	n.a.	n.a.	Breast cancer	Plasma	51	14	10	62	7
				72	76	n.a.	n.a.	Breast cancer	Plasma	45	37	27	39	
Ren [33]	2012	China	Asian	29	13	0.66	0.62	Pancreatic cancer	Fecal	25	4	4	9	6
Xie [27]	2012	China	Asian	45	30	0.62	0.57	Leukemia	Serum	41	6	4	24	4
Anjuman [28]	2013	USA	Caucasian/African	39	42	0.59	0.61	Lung cancer	Sputum	27	10	12	32	5
Zhao [34]	2013	France	Caucasian	68	42	0.68	0.52	Renal cancer	Serum	55	9	13	33	6
Iwamoto [5]	2014	Japan	Asian	34	23	0.76	0.48	Renal cancer	Serum	22	4	12	19	4
Li [35]	2014	USA	Caucasian/African	35	40	0.63	0.65	Lung cancer	Sputum	20	4	15	36	6
Shen [36]	2014	USA	Caucasian/African	64	73	0.64	0.66	Lung cancer	Sputum	43	18	23	50	5

n.a.: not available; TP: true positive; FP: false positive; FN: false negative; TN: true negative; QUADAS: quality assessment of diagnostic accuracy studies.
